# Considering the Appropriateness of the Factor Analytic Operationalization of Allostatic Load

**DOI:** 10.1097/PSY.0000000000000415

**Published:** 2016-11-01

**Authors:** Zander Crook, Tom Booth

**Affiliations:** Department of Psychology, The University of Edinburgh, Edinburgh, United Kingdom.; Department of Psychology, The University of Edinburgh, Edinburgh, United Kingdom.

In a recent issue of *Psychosomatic Medicine*, Wiley et al. (1) made a valuable contribution to the discussion of the optimal measurement of allostatic load (AL). In the most comprehensive factor analytic investigation of AL to date, they found that a bifactor model with a general AL factor and seven physiological system factors fits better than a higher-order model in which the seven system factors loaded on the general AL factor. Similar models have been applied by the author (T.B.) and others to operationalize AL (2–4). Here, we consider the primary theoretical assumptions underlying latent variable modeling, argue that the construct of AL is inconsistent with these assumptions, and propose alternate operationalizations of AL.

## UNDERLYING CONSTRUCT (COMMON CAUSE)

A latent variable model is estimated based on the patterns of covariance in a set of variables. By including an AL general factor in a latent variable model, researchers are positing that an underlying construct is the common cause of the observed covariation in all of the modeled biological measures. Although the theoretical relation of the common cause or construct to the original variables differs in bifactor versus higher-order models, in either case, we must ask: What could this common factor be? Wiley et al. stated that the AL factor “[captures] the notion that there is an underlying process influencing multiple physiological systems” ((1): p. 4). However, the observation of a general factor estimated from interindividual summary statistics (i.e., covariances) says little about what this process may actually be.

## INDEPENDENCE CONDITIONAL ON THE LATENT TRAIT

A primary assumption of latent variable models is that once the effect of the latent factors has been accounted for, the measured variables—in this case, the biological measures—are independent. This is unlikely to be the case with AL measures. Levels of different biomarkers are linked causally to each other, rather than only through the common cause latent variable(s). For example, body mass index (BMI) has previously been used as a metabolic system AL biological measure (e.g., (2,5)). However, Mendelian randomization studies have found that increased BMI has a causal effect on levels of other metabolic biological measures as well as levels of AL biomarkers used to represent other physiological systems, such as blood pressure and inflammation (e.g., (6)). Thus, it is most likely that the biomarkers are not conditionally independent but are instead dynamically related in complex networks. Such networks can produce observed correlations between variables that have no common cause (7).

## INTERCHANGEABILITY OF INDICATORS

A further assumption of the latent variable model is that the definition of the latent variable does not change when different sets of indicators are used (8). This holds because the indicators are affected by, but do not affect, the latent variable. Another key finding of Wiley et al. was that fitting models in which the biological measures from each of the 7 physiological systems were excluded caused no large changes in AL factor loadings (1). This method provides only a weak test of interchangeability. The stability of general intelligence factor loadings has long been a research focus for intelligence researchers, so AL researchers may benefit from applying their approaches to this issue (e.g., (9,10)). For example, researchers could compute and correlate AL scores from different nonoverlapping multisystem sets of biological measures (8). The existence of diverse causal links between AL biological measures from different physiological systems suggests to us that the nature of what relates the biomarkers may change depending on which measures are included in the model. We predict that more thorough, more powerful tests of the stability of AL factor loadings will find that it does not hold.

## FORMATIVE VERSUS REFLECTIVE INDICATORS

In the common factor model, the biological measures are reflective indicators, that is, they are manifested by a common cause latent variable. However, to the extent that the model assumptions are violated (previously discussed), the factor model is not appropriate. Thus, it may instead be profitable to consider the biological measures as formative indicators, that is, as variables that define the construct (8). This way of thinking about how the biological measures relate to AL is consistent with any number of weighted or sum scores. It is also consistent with AL theory, in that more severe, more widespread physiological dysregulation will relate to higher AL scores.

Alternatively, the associations between AL biological measures could be modeled using each measure individually, without the need for any single latent or observed summary. This could be done with network analysis, which has been used beneficially by researchers studying symptom networks in mental disorders (11). Allostatic load indicators can also be modeled separately without consideration of their associations. Consistent with this approach, *Psychosomatic Medicine* typically provides data of separate biological measures when articles report about complex phenomena such as AL and metabolic syndrome.

Aside from any issues regarding model assumptions, two further points warrant comment about the models presented by Wiley et al.

## IMPROVED MODEL FIT FOR BIFACTOR APPROACH

The complex causal links between biological measures from different physiological systems also help to explain why the bifactor AL model fits better than the hierarchical AL model. The hierarchical model imposes “proportionality constraints” ((12): p. 115): the ratio of the AL general factor loadings to the system factor loadings is constrained to equality within the biological measures of each physiological system. Considering the diverse causal links between different AL biomarkers, both within and across systems, these proportionality constraints are likely to be violated. Furthermore, it has been shown that when the true model contains “unmodelled complexity” ((13): p. 407) in the form of small correlated residuals and cross-loadings, or even modeled complexity in the form of correlated residuals across factors, fit indices and criteria may be biased in favor of the bifactor model. Consequently, the better fit of the bifactor model follows from AL theory and research, as well as from methodological findings, for reasons other than those Wiley et al. (1) focused on.

## VARIANCE EXPLAINED BY PHYSIOLOGICAL DYSREGULATION FACTORS

Statistically, a desirable property of a general factor is that it accounts for most variance in the constituent indicator variables. In the study by Wiley et al., the AL factor explained only approximately 11% of the variance in the AL biological measures. Some of the physiological system-specific factors were also weak. For example, the hypothalamic-pituitary-adrenal axis and inflammation factors explained only approximately 9% and 16% of the variance in their respective biological measures. Note that weak factor saturation of physiological dysregulation factors has also been an issue in other samples (2,3).

## PROPERTIES OF OPTIMAL SCORES FOR AL

Ideally, AL scores should be: 1) calculated using biological measures from various physiological systems; 2) consistently calculated across samples; and (3) closely related to criterion variables. Those who desire scores that are rooted in AL theory would prefer the AL scoring method that produces the scores most closely related to chronic/repeated perceived stress. For a pragmatist, the focus may not be on investigating how different physiological dysregulation scores relate to prior perceived stress but rather on finding the scores that most strongly predict important health outcomes such as cardiovascular disease and death. It may also be advantageous to have scores that explicitly represent the accumulation of the effects of repeated environmental challenges.

Our theoretical and methodological concerns with the factor analytic operationalization of AL suggest to us that factor scores will not prove to be the optimal AL scoring method. We therefore believe that further research is required to determine the optimal operationalization(s) of AL.

**ZANDER CROOK, MSc**
Department of Psychology
The University of Edinburgh, Edinburgh
United Kingdom **TOM BOOTH, PhD**
Department of Psychology
The University of Edinburgh, Edinburgh
United Kingdom
